# The Hospital Crisis Once More

**Published:** 1920-11-13

**Authors:** 


### The Hospital

The Workers' Newspaper of Administrative Medicine and Institutional Life, Administration, National Insurance and Health.

No. 1797, Vol. LXIX. SATURDAY, NOVEMBER 13, 1920. PRICE TWOPENCE

### THE HOSPITAL CRISIS ONCE MORE.

While we have the greatest respect for Dr. Addison, we have come to the conclusion that if he ever had a clear understanding about voluntary hospital finance, it has been crowded out of his mind by his many other important preoccupations. During the second reading of the Miscellaneous Powers Bill he informed the House of Commons that hospitals might relieve pressing financial duties by making use of free legacies to meet current expenditure. As most hospitals have for many years past spent every penny of money received from this source on current expenditure—a step they are quite entitled to take—it cannot be claimed that the suggestion is going to help greatly. Possibly the Minister means that hospitals should spend accumulated free legacies, if any, but as here again in most cases any investments that have no trust attached to them have long since been earmarked as security for a banker's loan, this is an equally unsuitable solution to the problem. We think that it would be a good move if Dr. Addison had upon his staff an assistant, with a practical inside knowledge of hospital conditions, who could advise him on technical points.

In respect of income of hospitals from other sources the Minister of Health is also badly informed. The aggregate of donations and subscriptions has not decreased; in fact, it is more likely that it has materially increased. The whole trouble lies in the huge cost of maintenance, an expense so tremendous that temporarily, at least, it seriously embarrasses the hospital system and entirely delays necessary evolution in directions indicated by modern medical and surgical science.

With quite a proprietary air Dr. Addison mentions the emergency fund of £250,000 distributed by King Edward's Hospital Fund for London and the £700,000 from the National Relief Fund, and says that in respect of the distribution of the last a body is now in being. Repeated mention of these sums, which any time during the past many weeks the Ministry of Health have brought round and round again to produce the illusion of a stage army, is in our opinion driving away support from the hospitals. Our respected contemporary, the *Daily News*, has a very short memory, and comes forward on the strength of the *nth* announcement, with the headlines "Big Windfall for Hospitals. Million Pounds to Relieve Financial Distress." In any case, if the million were multiplied in reality, as it has been by illusion, the sum would be but a drop in the bucket of hospital requirements.

There is no doubt that what is called the hospital crisis is not over. Lord Sandhurst has told us that the substantial sum raised by St. Bartholomew's Hospital through an unusually vigorous appeal has had to be spent for current needs, and, if bad times continue (which means if the high cost of living prevails), the great City hospital must raise an equally large sum every year. St. Thomas's, King's College, St. George's, the London, and many other famous institutions are in like predicament. The larger the hospital, as a rule, the larger the need, but we are glad to say that none has lost courage and thrown up the sponge. Viscount Knutsford, speaking at the meeting of the Federation of Medical and Allied Services, said: "My belief is that the voluntary system has come to an end. I declare to you that on January 1 the London Hospital cannot afford to pay its bills—we must close. We simply cannot afford it." His explanation of the cause of the trouble is identical with ours, as stated above; his proposed method of meeting the situation is different. But in passing let it be recorded that the London Hospital is receiving from £50,000 to £100,000 a year short of its requirements. Mr. Morris, the House Governor, is more cheerful than his Chairman. He has informed the press that Lord Knutsford's announcement must be regarded as an expression of his own opinion only. "Not only shall we not close on January 1," he says, "but I hope we shall not close at all. . . . While there are beds we never shall shut the gates, but if income goes dwindling down the time may come when the hospital would be nothing more than a Poor-law institution." Not, as he describes it, an institution where teaching, research and consultative treatment rank as high as the curing of the sick, but a retrograde place, such as the spittal of olden days, where all that was given was warmth, food, and some physic and surgery to a limited number of the more desperate cases.

We thank Mr. Morris for his repellent picture. We wish the public to see the situation in its whole detail, and realise that it is not the physical needs of a comparative few that are at stake, but the health also of our children and children's children, and the efficiency and prosperity of a nation.

We believe that the public can and will respond. We believe that they will not respond the less if the State adequately recognises its responsibilities in respect of payment for such public services as the education of doctors and nurses, research, and the treatment of patients, which by statute has become an affair beyond the scope of voluntary effort.

Certainly it is a fight against time. Each day increases the difficulties, but each day matures judgment and ripens schemes for amelioration. It is too early yet accurately to value the new branch of income provided by patients' payments. The result of the huge Red Cross effort is still unknown to us. The process of broadening the basis of hospital revenue by means of workpeople's weekly contributions and house-to-house collections is still in its early days. The insurance companies have not recognised their advantages and responsibilities in respect of hospitals. Busy brains are evolving schemes which will press but lightly upon the multitude, yet in the aggregate bring in handsome revenue. It is a time of hope rather than of despair.

But let the public know all. Let us show them that they have a precious possession, and must not through neglect endanger this great heritage, not merely by the possibility of the deterioration of the standard it has now attained, but by the stinting of means to ensure its advancement to that higher stage of scientific perfection which is its destiny and just reward.

